# Neonatal jaundice and the infant gut microbiome: an integrated shotgun metagenomics and bidirectional Mendelian randomization study in Xinjiang

**DOI:** 10.3389/fmicb.2026.1761712

**Published:** 2026-02-26

**Authors:** Muqing Niu, Jinyong Pan, Yan Guo, Fengling Zhang, Hua Guan, Xiaoping Yang, Hu Li, Heyun Xiong, Yan Zhang, Yonglin Chen

**Affiliations:** 1 The First Affiliated Hospital of Shihezi University, Shihezi, Xinjiang, China; 2 Department of Pediatrics, The First Affiliated Hospital of Shihezi University, Shihezi, Xinjiang, China; 3 Shihezi University School of Medicine, Shihezi, Xinjiang, China; 4 Clinical Medical Research Center for Children's Diseases in the Xinjiang Production and Construction Corps, Shihezi, Xinjiang, China; 5 Fourth Division Hospital of the Xinjiang Production and Construction Corps, Kokdala, Xinjiang, China; 6 Fifth Division Hospital of the Xinjiang Production and Construction Corps, Shuanghe, Xinjiang, China

**Keywords:** bile acid metabolism, carbohydrate-active enzymes, gut microbiome, infant, Mendelian randomization, microbiome-based therapy, neonatal jaundice, shotgun metagenomics

## Abstract

**Background:**

Neonatal jaundice is a common condition, yet inter-individual variation in its onset and severity cannot be fully explained by traditional clinical risk factors. Emerging evidence suggests that the infant gut microbiome may modulate bilirubin metabolism, but its compositional and functional signatures in jaundiced neonates remain incompletely defined. This study aimed to characterize the taxonomic and functional features of the gut microbiome in neonatal pathologic jaundice and to explore potential causal links using Mendelian randomization (MR).

**Methods:**

We conducted a case–control study of term infants with pathologic jaundice and matched healthy controls. Stool samples were subjected to shotgun metagenomic sequencing to assess microbial diversity, taxonomic composition, functional gene repertoires, and carbohydrate-active enzyme families, and publicly available genome-wide association study summary statistics were used to perform bidirectional MR between microbiome-related traits and neonatal jaundice.

**Results:**

Alpha diversity indices did not differ significantly between groups, whereas beta diversity based on Bray–Curtis dissimilarity showed clear separation of jaundiced and control infants, indicating a restructured microbial community rather than a simple loss of richness. Jaundiced neonates exhibited increased relative abundance of Gram-negative taxa, including *Escherichia coli*, and reduced levels of putatively beneficial genera such as *Bifidobacterium* and *Lactobacillus*. Functionally, pathways involved in bile acid synthesis and metabolism, carbohydrate and energy metabolism, and cofactor and vitamin biosynthesis were enriched in the jaundiced group, accompanied by marked shifts in carbohydrate-active enzyme profiles. Forward MR suggested that several microbial metabolic pathways exert genetically predicted effects on jaundice risk, whereas reverse MR provided little evidence that genetic liability to jaundice substantially alters microbiome traits.

**Conclusions:**

Neonatal pathologic jaundice is associated with distinctive compositional and functional alterations in the gut microbiome. Genetic evidence from MR supports a potential causal contribution of specific microbial pathways to jaundice risk, highlighting candidate targets for microbiome-based prevention or adjunctive therapy in early life.

## 1 Introduction

Neonatal jaundice (NJ) is a common condition that affects the majority of newborns worldwide, with approximately 60%−80% of infants exhibiting some degree of jaundice. While most cases are physiological and resolve without treatment, pathological jaundice can lead to severe complications such as kernicterus, which results in long-term neurological damage (Olusanya et al., 2018). Although the exact mechanisms behind NJ are not fully understood, emerging evidence suggests that gut microbiota may play a key role in bilirubin metabolism, potentially influencing the development and severity of jaundice (Su et al., 2024).

The human gut microbiome, especially during infancy, is a dynamic and complex community that significantly impacts host metabolism, immune function, and overall health (Arrieta et al., 2014). Recent studies have highlighted that certain gut bacteria may modulate processes such as bile acid metabolism and glucuronidation, which are critical in the breakdown and excretion of bilirubin (Lin et al., 2025). Despite these observations, the causal relationship between gut microbiota and NJ has not been definitively established, leaving an important gap in our understanding of the disease (Chen and Yuan, 2020).

This study combines shotgun metagenomics and bidirectional Mendelian randomization (MR) to explore the relationship between neonatal jaundice and the infant gut microbiome (Davey Smith and Hemani, 2014). Shotgun metagenomics provides a comprehensive analysis of microbial diversity and functional capacity (Sharpton, 2014), while MR allows for the identification of causal relationships by leveraging genetic data. Our research aims to (1) compare the gut microbiota composition in jaundiced and healthy neonates; (2) examine the causal link between NJ and gut microbiota; and (3) identify microbial pathways involved in bile acid metabolism that may contribute to jaundice. Conducted in Xinjiang, a region with distinct environmental and dietary factors (Tao et al., 2022), this study provides new insights into the microbiome's role in NJ and its potential as a target for therapeutic interventions.

## 2 Materials and methods

This study integrated shotgun metagenomic sequencing and bidirectional Mendelian randomization to characterize gut microbiome alterations in neonatal jaundice and to explore potential causal relationships. An overview of the study design and analytical workflow is provided in Supplementary Figure S1.

### 2.1 Metagenomics

#### 2.1.1 Study population and sample collection

This study included neonates from the Xinjiang region, with 11 jaundice-affected neonates and five healthy controls. Neonates in the jaundice group were diagnosed with neonatal jaundice within 48 h of birth, while the control group consisted of healthy neonates without any jaundice or major health conditions (Kemper et al., 2022). Fecal samples were collected non-invasively using sterile containers within 48 h of birth and immediately stored at −80 °C for DNA extraction and analysis (Wu et al., 2019). Neonates with congenital diseases or those unable to provide a sample were excluded from the study.

#### 2.1.2 Fecal DNA extraction and library preparation

Genomic DNA was extracted from each fecal sample using the CTAB method following standardized procedures (Human Microbiome Project Consortium, 2012). DNA concentration and integrity were verified using a Qubit^®^ fluorometer and agarose gel electrophoresis (Versmessen et al., 2024). A total of 1 μg of high-quality DNA was fragmented to an average size of approximately 350 bp using a Covaris ultrasonicator (Quince et al., 2017).

Sequencing libraries were prepared using the NEBNext^®^ Ultra DNA Library Prep Kit for Illumina, involving end-repair, A-tailing, adapter ligation, size selection, and PCR enrichment (Tvedte et al., 2021). Library fragment size distribution was assessed using the AATI system, and the effective library concentration was quantified by qPCR. Libraries with concentrations >3 nM were pooled and subjected to next-generation sequencing (Robin et al., 2016).

#### 2.1.3 Shotgun metagenomic sequencing

Metagenomic sequencing was performed on the Illumina NovaSeq 6000 platform using a paired-end 150 bp (PE150) strategy (Modi et al., 2021). Each sample generated approximately 8.2–11.6 Gb of raw sequencing data (Jiménez-Arroyo et al., 2025). All sequencing procedures were conducted by a commercial sequencing provider according to standard operating protocols.

#### 2.1.4 Quality control and removal of host sequences

Raw sequencing reads were processed using fastp for quality filtering, during which paired reads containing adapter sequences (Chen et al., 2018), reads with more than 50% low-quality bases (*Q* ≤ 5), and reads with over 10% ambiguous nucleotides were removed (Song et al., 2023). Both forward and reverse reads were filtered independently (Schmieder and Edwards, 2011).

To remove host-derived contamination, the clean reads were aligned against the human reference genome (GRCh38) using Bowtie2 with the parameters –end-to-end, –sensitive, -I 200, and -X 400 (Langmead and Salzberg, 2012). Reads that did not map to the host genome were retained as non-host microbial reads for downstream metagenomic analyses.

#### 2.1.5 Metagenomic assembly, gene prediction, and construction of a nonredundant gene catalog

High-quality non-host reads were assembled *de novo* using MEGAHIT with the meta-large preset optimized for complex microbial communities (Li et al., 2015). The resulting scaffolds were split at ambiguous bases to generate continuous scaftigs (Jin et al., 2024), and fragments shorter than 500 bp were removed (Uritskiy et al., 2018). Open reading frames (ORFs) were predicted from scaftigs using MetaGeneMark with default parameters. ORFs shorter than 100 nucleotides were discarded (Al-Ajlan and El Allali, 2018). To build the initial gene catalog, predicted genes were clustered using CD-HIT with a sequence identity threshold of 95% and coverage threshold of 90% (Li and Godzik, 2006). Clean reads were then mapped back to the gene catalog using Bowtie2 to calculate gene-level read counts (Langmead and Salzberg, 2012). Genes supported by ≤ 2 mapped reads were removed. Gene abundance was normalized through gene length and total mapped reads (Uritskiy et al., 2018).

#### 2.1.6 Taxonomic annotation

Taxonomic profiling was performed by aligning the nonredundant gene set against the microbial subset of the NCBI NR database (Micro_NR) using DIAMOND blastp with an e-value cutoff of 1e−5 (Buchfink et al., 2015). Taxonomic assignments were determined using the lowest common ancestor (LCA) algorithm (Huson et al., 2016). Gene abundances annotated to each taxon were summed to generate taxonomic abundance profiles from the phylum to species levels.

Downstream taxonomic analyses included relative abundance visualization, hierarchical clustering heatmaps, Krona plots (Ondov et al., 2011), principal component analysis (PCA), principal coordinates analysis (PCoA), and non-metric multidimensional scaling (NMDS). Beta-diversity group differences were tested primarily using PERMANOVA (adonis2, permutations = 9,999), and homogeneity of multivariate dispersion was assessed using PERMDISP (betadisper/permutest). ANOSIM results were provided as supplementary confirmation (Kustrimovic et al., 2023).

#### 2.1.7 Functional annotation

Functional profiling was conducted by aligning genes to several curated functional databases using DIAMOND, including: Kyoto Encyclopedia of Genes and Genomes (KEGG), eggNOG orthologous groups, Carbohydrate-Active Enzymes (CAZy), Virulence Factor Database (VFDB), Pathogen–Host Interaction database (PHI-base). For each gene, the best-scoring hit was used for annotation. Functional abundance matrices were generated by summing abundances across annotated genes. Analyses included functional composition profiling, heatmap clustering, PCA, NMDS, and pathway-level comparisons based on KEGG modules and pathways (Quince et al., 2017).

#### 2.1.8 Antibiotic resistance genes and mobile genetic elements

Antibiotic resistance genes (ARGs) were identified using the Comprehensive Antibiotic Resistance Database (CARD) via the RGI tool with a strict threshold (e-value < 1e−30). ARG abundances were derived by combining gene-level abundance values. Mobile genetic elements (MGEs)—including insertion sequences, integrons, and plasmids—were annotated using DIAMOND against corresponding reference databases and visualized through barplots, heatmaps, and multivariate analyses (Siguier et al., 2006).

#### 2.1.9 Diversity analysis

Alpha diversity was assessed using Shannon, Simpson, Chao1, and observed species indices. Beta diversity was evaluated using Bray–Curtis dissimilarity followed by PCoA and NMDS ordination. Group-level differences in microbial community structure and functional composition were tested using PERMANOVA (adonis2, permutations = 9,999), and multivariate dispersion was assessed using PERMDISP (betadisper/permutest) to ensure that PERMANOVA results were not driven by unequal within-group dispersion. ANOSIM was used as a supplementary test (Lozupone and Knight, 2005).

#### 2.1.10 Differential abundance analysis

Group-level differences in microbial taxa and functional features were analyzed using the MetaStat and MetaGenomeSeq frameworks. The metagenomeSeq fitZIG model was applied to detect differential taxa and pathways, while the Wilcoxon rank-sum test was used for pairwise comparison. LEfSe was employed for biomarker discovery with an LDA score threshold of 4. *p*-values were adjusted for multiple testing using the Benjamini–Hochberg procedure when appropriate.

Random forest classification models were constructed using species-level abundance profiles to identify discriminative microbial signatures. Model performance was evaluated through 10-fold cross-validation and receiver operating characteristic curves (Segata et al., 2011).

#### 2.1.11 Software and computational environment

All bioinformatics analyses were performed using standard open-source tools, including fastp, Bowtie2, MEGAHIT, MetaGeneMark, CD-HIT, DIAMOND, MetaGenomeSeq, and LEfSe. Statistical analyses and visualizations were conducted in R (version 4.4.1) using the tidyverse, vegan, pheatmap, ade4, randomForest, and pROC packages. All computations were executed on a Linux-based high-performance computing environment.

### 2.2 Mendelian randomization

#### 2.2.1 Data sources and selection of instrumental variables

This study utilized the Mendelian Randomization (MR) method to explore the causal relationship between neonatal jaundice and gut microbiota (Burgess et al., 2013). The data we used came from two primary sources: the FinnGen project and the IEU OpenGWAS (Sun et al., 2024).

For forward MR (microbiome as exposure and neonatal jaundice as outcome), gut microbiome traits (taxa and functional pathway abundance traits) were treated as exposures and neonatal jaundice was treated as the outcome. For reverse MR (neonatal jaundice as exposure and microbiome traits as outcomes), neonatal jaundice was treated as the exposure and each microbiome trait was treated as the outcome.

Neonatal jaundice summary statistics were obtained from the FinnGen project, specifically the P16_NEONTAL_JAUND_OTH_UNSP_CAUSES phenotype, which represents neonatal jaundice of other and unspecified causes (Kurki et al., 2023). This phenotype has been analyzed across multiple FinnGen releases and includes a large number of individuals from the Finnish population. Gut microbiome GWAS summary statistics were obtained from the IEU OpenGWAS database and included multiple datasets describing both microbial taxa and functional pathway abundance traits. For example, the dataset ebi-a-GCST90027449 comprises 7,738 samples and provides GWAS summary statistics for gut microbiome functional pathways (Lopera-Maya et al., 2022). These summary statistics were used as exposures or outcomes depending on the direction of the MR analysis.

To conduct the MR analyses, appropriate instrumental variables (IVs) were selected. Single nucleotide polymorphisms (SNPs) were required to be strongly associated with the exposure of interest and independent of potential confounders of the exposure–outcome relationship. Specifically, SNPs associated with the exposure at a significance threshold of *p* < 1 × 10^−5^ were selected as candidate IVs, thereby ensuring sufficient instrument strength and minimizing bias due to weak instruments (Pierce et al., 2011).

#### 2.2.2 Statistical analysis

The main objective of the Mendelian Randomization analysis in this study was to evaluate the causal relationship between neonatal jaundice and gut microbiota. We employed several MR methods, including the Inverse Variance Weighted (IVW) method, MR Egger regression, Simple Mode, and Weighted Median (Bowden et al., 2016), to ensure the robustness and consistency of the results.

Inverse variance weighted (IVW): this is the most commonly used weighted regression method in MR analysis, which weights each instrumental variable by its effect size and standard error to provide an overall effect estimate of the exposure-outcome relationship.

MR Egger regression: this method is used to assess whether there is bias in the instrumental variables, such as horizontal pleiotropy. MR Egger regression estimates the causal effect between the exposure and outcome and provides bias detection (Bowden et al., 2015).

Simple mode and weighted median: these two methods estimate the causal effect between exposure and outcome using different weightings, particularly providing robust estimates when some instrumental variables are biased (Hartwig et al., 2017).

To assess statistical significance, we used the inverse-variance weighted (IVW) method as the primary estimator (Wald ratio for traits instrumented by a single SNP). Given the large number of microbial traits tested, we controlled for multiple comparisons using the Benjamini–Hochberg false discovery rate (BH-FDR) across all tested traits within each MR direction (forward and reverse). Associations with BH-FDR *q* < 0.05 were considered statistically significant, while nominal *p* < 0.05 results were treated as suggestive and interpreted cautiously.

All statistical analyses were performed in R (version 4.4.1; R Foundation for Statistical Computing, Vienna, Austria) using the TwoSampleMR package. Standard MR sensitivity analyses (Cochran's *Q* for heterogeneity, MR-Egger intercept for directional pleiotropy, leave-one-out analysis, and MR-PRESSO where applicable) should be reported for both forward and reverse MR to evaluate core MR assumptions (Hemani et al., 2018).

## 3 Results

### 3.1 Differences in the composition of the gut microbiota

**Figure 1:**
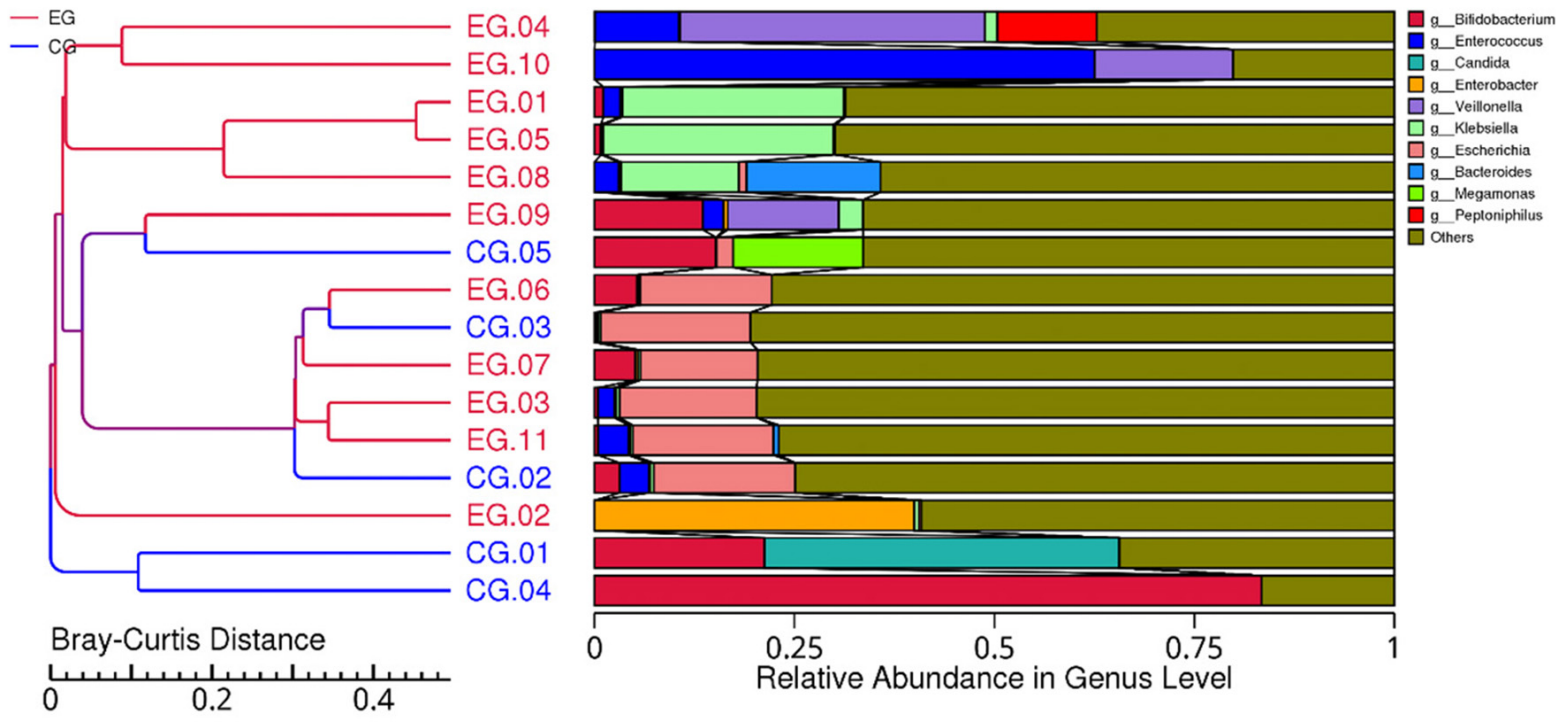
Relative abundance of the top 10 genera in the neonatal gut microbiome across jaundiced and healthy infants. Bars represent the proportion of each genus within individual samples, with colors indicating taxonomic groups.

Across all samples, shotgun metagenomic sequencing generated high-quality microbial profiles suitable for taxonomic and functional analyses. Taxonomic profiling revealed that the neonatal gut microbiome was dominated by the phyla Firmicutes, Proteobacteria, Actinobacteria, and Bacteroidetes, consistent with early-life microbial colonization patterns. At the genus level, *Escherichia, Enterococcus, Bifidobacterium*, and *Streptococcus* were among the most abundant taxa in both groups (Figure 1).

Comparative analyses demonstrated distinct microbial signatures between neonates with jaundice and healthy controls. Several taxa showed group-specific shifts in relative abundance, indicating altered microbial colonization in infants with elevated bilirubin levels. Alpha diversity indices (Shannon, Simpson, Chao1, and observed species) showed no significant difference between groups, suggesting comparable overall microbial richness and evenness.

**Figure 2:**
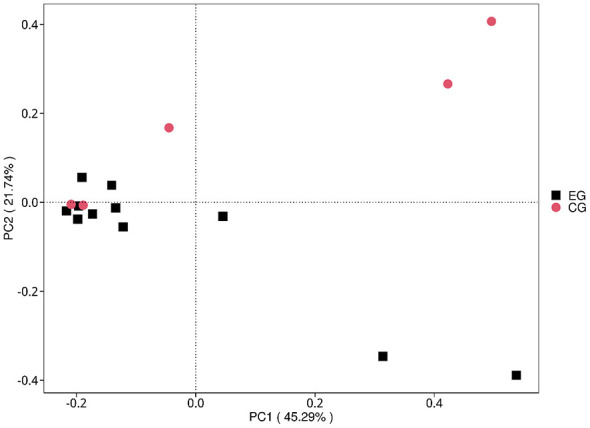
Principal coordinates analysis (PCoA) based on Bray–Curtis dissimilarity at the genus level. Each point represents one sample; colors denote jaundiced and healthy groups. PERMANOVA (Bray–Curtis) was used to test group differences (pseudo-*F* = 1.773, *R*^2^ = 0.112, *p* = 0.1029; 9,999 permutations), and multivariate dispersion was assessed using PERMDISP (*F* = 1.144, *p* = 0.4337).

In contrast, beta diversity analyses suggested separation between jaundice and control samples. Both principal coordinates analysis (PCoA) and non-metric multidimensional scaling (NMDS) based on Bray–Curtis dissimilarity showed group-wise patterning (Figure 2). This pattern was evaluated by PERMANOVA (Bray–Curtis: pseudo-*F* = 1.773, *R*^2^ = 0.112, *p* = 0.0968; 9,999 permutations). Homogeneity of dispersion was not significantly different between groups (PERMDISP: *F* = 1.144, *p* = 0.4397), supporting that the observed ordination pattern was not driven by unequal dispersion.

### 3.2 Analysis of the abundance differences in the functional genes of the gut microbiota

**Figure 3:**
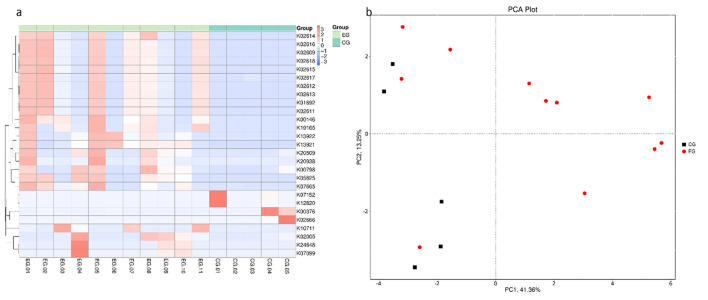
KEGG ortholog (KO)–based functional clustering and PCA analysis between jaundiced (EG) and healthy (CG) neonates. **(a)** Heatmap of differentially abundant KEGG KOs across all samples. Rows represent KO identifiers and columns represent individual samples. Colors indicate *Z*-score–normalized abundances. Hierarchical clustering of both samples and KOs demonstrates distinct functional signatures between EG and CG groups. **(b)** Principal component analysis (PCA) based on KO abundance profiles. Each point represents one sample; red circles indicate EG and black squares indicate CG. The separation along PC1 and PC2 reflects differences in microbial functional structure associated with neonatal jaundice. For completeness, beta-diversity on KO profiles was evaluated using Bray–Curtis PERMANOVA (pseudo-*F* = 1.637, *R*^2^ = 0.105, *p* = 0.1369; 9,999 permutations) with dispersion assessed by PERMDISP (*F* = 3.350, *p* = 0.2046).

Functional annotation using KEGG, eggNOG, CAZy, VFDB, and PHI databases revealed substantial differences in functional gene profiles between the two groups. As shown in Figure 3, at the KEGG ortholog (KO) and pathway levels, infants with jaundice exhibited distinct patterns in genes related to carbohydrate metabolism, amino acid metabolism, energy metabolism, and cofactor/vitamin biosynthesis.

**Figure 4:**
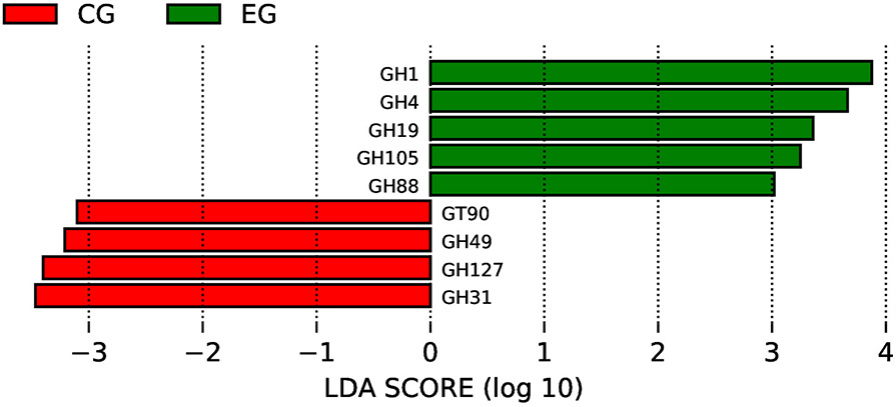
Differential CAZy Level 2 enzyme families identified by LEfSe analysis between jaundiced (EG) and healthy (CG) neonates. The LDA (log_10_) bar plot shows carbohydrate-active enzyme (CAZy) families with significantly different abundances between groups. Green bars represent enzyme families enriched in the EG group (e.g., GH1, GH4, GH19, GH105, GH88), whereas red bars represent families enriched in the CG group (GT90, GH49, GH127, GH31). LDA scores indicate the effect size of each differentially abundant feature.

Carbohydrate-active enzyme (CAZy) profiling identified significant differences in glycoside hydrolases, glycosyltransferases, and carbohydrate-binding modules between the two groups (Figure 4). CAZy-based NMDS and clustering analyses further confirmed the functional divergence in carbohydrate metabolism between jaundiced and healthy neonates.

Virulence factor annotation (VFDB) showed group-specific variations in functional categories associated with adhesion, invasion, secretion systems, and stress response. PHI-base annotation similarly revealed differential enrichment of genes involved in pathogen–host interaction, suggesting altered functional potential linked to microbial adaptation and host interactions.

**Figure 5:**
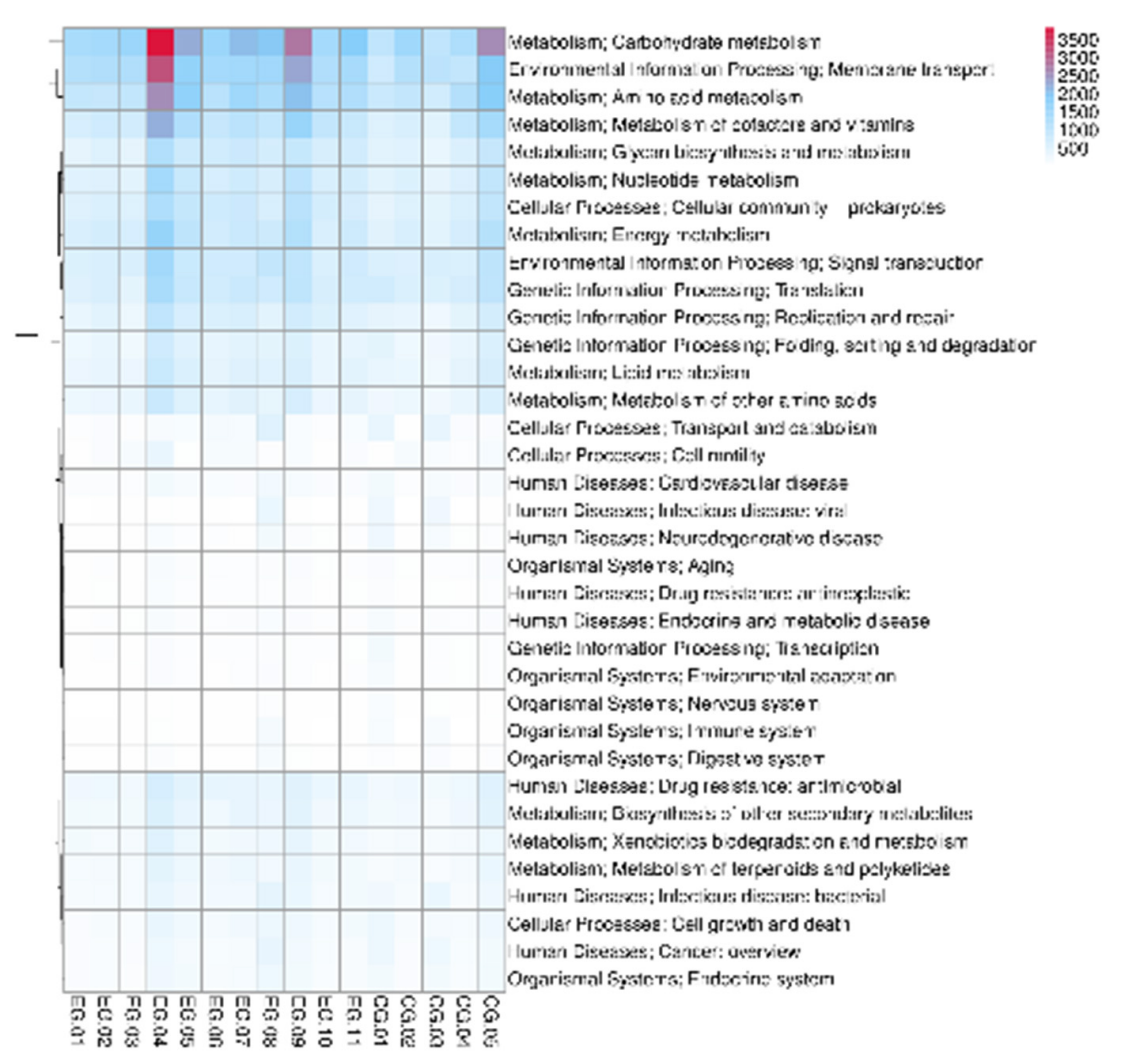
Heatmap and hierarchical clustering of KEGG Level 2 functional categories across neonatal fecal samples. The heatmap displays *Z*-score–standardized relative abundances of KEGG Level 2 metabolic and cellular functional categories. Columns represent individual samples, and rows represent functional pathways. Hierarchical clustering of both samples and functional categories reveals group-specific functional patterns between jaundiced (EG) and healthy (CG) infants, particularly in pathways related to carbohydrate metabolism, membrane transport, amino acid metabolism, and energy metabolism.

Differential abundance testing using MetaGenomeSeq and Wilcoxon rank-sum methods identified multiple significantly enriched functional genes in neonates with jaundice, indicating shifts in microbial metabolic capacity and physiological function during early colonization. Hierarchical clustering of KEGG Level 2 functional categories further highlighted group-specific functional patterns between jaundiced and healthy infants (Figure 5).

### 3.3 Significant differences in functional metabolic pathways

**Figure 6:**
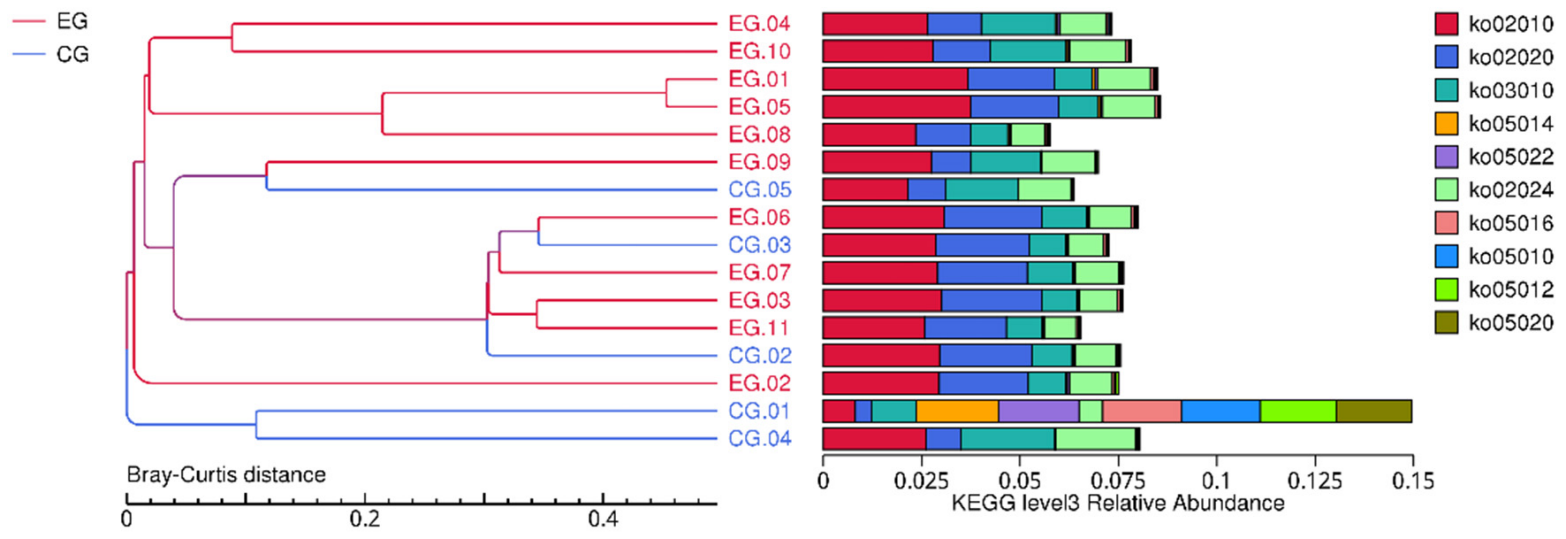
KEGG pathway barplot. Relative abundance of the top KEGG Level 3 pathways across samples. Bars represent pathway composition profiles, illustrating distinct metabolic functions associated with neonatal jaundice.

KEGG pathway–level comparisons revealed distinct metabolic patterns between groups (Figure 6). Neonates with jaundice showed altered abundance of pathways associated with carbohydrate metabolism, lipid metabolism, amino acid utilization, ABC transporters, and two-component regulatory systems. Several pathways related to energy production, oxidative stress, and bile acid transformation displayed group-specific differences.

**Figure 7:**
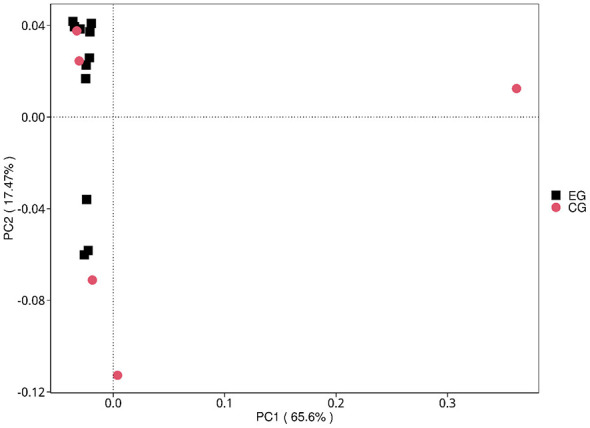
KEGG Level 3 PCoA. PCoA plot based on KEGG Level 3 pathway profiles (Bray–Curtis). Group differences were tested by PERMANOVA (pseudo-*F* = 2.086, *R*^2^ = 0.130, *p* = 0.0661; 9,999 permutations). Dispersion was assessed using PERMDISP (*F* = 9.161, *p* = 0.0642).

Hierarchical clustering of KEGG pathway relative abundances separated jaundice and control samples, suggesting metabolic pathway-level divergence. Pathway-level ordination analyses (PCoA/NMDS) were consistent with this pattern (Figure 7). PERMANOVA provided evidence for a trend toward global functional separation at KEGG Level 3 (Bray–Curtis: pseudo-*F* = 2.086, *R*^2^ = 0.130, *p* = 0.0661; 9,999 permutations). PERMDISP indicated a borderline difference in dispersion (*F* = 9.161, *p* = 0.0625), therefore the ordination should be interpreted cautiously.

Further pathway enrichment analysis identified modules that were significantly overrepresented or underrepresented in jaundiced neonates, reflecting functional alterations in core microbial metabolic networks.

### 3.4 Mendelian randomization–based genetic analysis and sensitivity validation in neonatal jaundice

**Figure 8:**
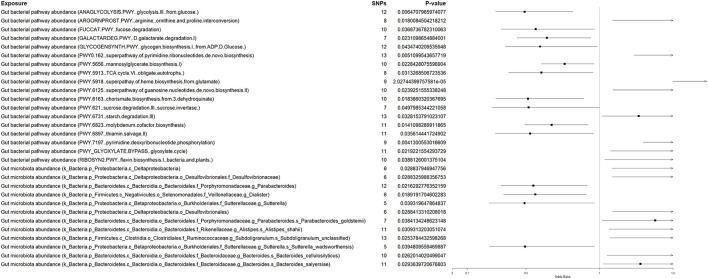
Forward Mendelian randomization analysis of gut microbiome features on neonatal jaundice. Forest plot displaying odds ratios (ORs) and 95% confidence intervals for the associations between genetically predicted gut microbial functional pathways and taxonomic traits (exposures) and the risk of neonatal jaundice (outcome), estimated using the inverse variance weighted method. Only the heme biosynthesis superpathway (PWY.5918) remained statistically significant after Benjamini–Hochberg false discovery rate (BH-FDR) correction, while other associations represent nominally significant or suggestive signals. The number of instrumental SNPs for each trait is indicated. Full MR results, including *p*-values and BH-FDR–adjusted *q*-values, are provided in Supplementary Table S1.

Using Mendelian randomization, we evaluated the genetically predicted effects of 411 gut microbial traits on neonatal jaundice in the forward direction (Figure 8). After correction for multiple testing using the Benjamini–Hochberg false discovery rate (BH-FDR) across all tested traits, only the heme biosynthesis superpathway (PWY.5918) remained statistically significant (IVW: OR = 2.755, 95% CI: 1.729–4.390; *p* = 2.0 × 10^−5^; *q* = 0.0083). This result suggests that a genetically predicted higher abundance of this functional pathway may be associated with an increased risk of neonatal jaundice. Several additional functional pathways showed nominal associations (*p* < 0.05), including pyrimidine ribonucleotide biosynthesis (PWY0.162), anaerobic glycolysis (ANAGLYCOLYSIS.PWY), molybdenum cofactor biosynthesis (PWY.6823), and starch degradation (PWY.6731); however, none of these associations survived FDR correction (all *q* > 0.05) and were therefore considered suggestive rather than definitive.

At the taxonomic level, several microbial traits demonstrated nominal associations in the forward MR analyses. Genetically predicted higher abundance of Proteobacteria-related lineages, including Deltaproteobacteria and related Desulfovibrionales taxa, was associated with an increased risk of neonatal jaundice, whereas Sutterella (a Burkholderiales lineage) showed an inverse association. As none of these taxonomic associations remained significant after FDR correction, they were treated as exploratory signals, and no direct causal attribution between specific taxa and functional pathways was inferred.

**Figure 9:**
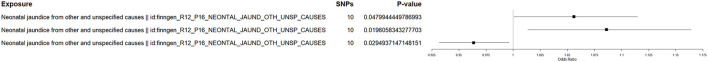
Reverse Mendelian randomization analysis of neonatal jaundice on gut microbiome traits. Forest plot showing odds ratios (ORs) and 95% confidence intervals for the effects of genetically predicted liability to neonatal jaundice (exposure) on gut microbial functional pathways and taxonomic traits (outcomes), estimated using the inverse variance weighted method. Several associations reached nominal significance; however, none remained statistically significant after Benjamini–Hochberg false discovery rate (BH-FDR) correction, indicating limited evidence for reverse causality. The number of instrumental SNPs for each trait is indicated. Full MR results, including *p*-values and BH-FDR–adjusted *q*-values, are provided in Supplementary Table S1.

In the reverse direction (Figure 9), we assessed whether genetic liability to neonatal jaundice influenced gut microbial traits (n = 412 traits). Three traits reached nominal significance (*p* < 0.05), including starch degradation III (PWY.6731), Burkholderiales, and methylphosphonate degradation I (PWY0.1533). However, none of these associations survived BH-FDR correction (all *q* > 0.05), providing limited evidence to support reverse causality from neonatal jaundice to gut microbiome composition or function.

Full Mendelian randomization results from the primary inverse variance weighted and Wald ratio analyses, including effect estimates, *p*-values, and BH-FDR–adjusted *q*-values for all tested traits, are provided in Supplementary Table S1. Details of the genetic instruments used in the forward and reverse Mendelian randomization analyses are summarized in Supplementary Table S2. Sensitivity analyses evaluating key Mendelian randomization assumptions—including instrument strength assessed using exposure-level *F*-statistics, heterogeneity evaluated by Cochran's *Q* statistics, horizontal pleiotropy assessed using the MR-Egger intercept and MR-PRESSO where applicable, and leave-one-out diagnostics—are presented in Supplementary Table S3 and the accompanying supplementary figures.

## 4 Discussion

Our study demonstrates that neonates with jaundice harbor a distinct gut microbiota configuration compared with healthy controls. Although alpha diversity indices did not differ significantly between groups, beta diversity analyses based on Bray–Curtis dissimilarity revealed a clear separation between jaundiced and control infants, indicating a restructured microbial community rather than a simple loss of richness (You et al., 2023). Consistent with previous reports on early-life dysbiosis (Dong et al., 2018), jaundiced neonates showed an increased relative abundance of Gram-negative bacteria such as *Escherichia coli*, together with reduced levels of putatively beneficial genera including *Bifidobacterium* and *Lactobacillus*. This shift toward a more pro-inflammatory and β-glucuronidase-producing community is biologically plausible in the context of hyperbilirubinemia: *E. coli* can deconjugate bilirubin via β-glucuronidase and thereby promote enterohepatic recirculation of unconjugated bilirubin, whereas bifidobacteria and lactobacilli have been reported to enhance intestinal barrier function and facilitate bilirubin excretion (Liu et al., 2024). Taken together, our compositional findings support the hypothesis that neonatal jaundice is accompanied by a characteristic pattern of gut microbial imbalance.

Beyond taxonomic alterations, we observed marked differences in microbial functional capacity between jaundiced and healthy neonates. Metagenomic profiling revealed enrichment of pathways involved in bile acid synthesis and metabolism, carbohydrate and energy metabolism, and cofactor and vitamin biosynthesis in the jaundiced group. Dysregulation of bile acid–related pathways is particularly relevant, as bile acids play a central role in regulating bilirubin solubility, transport, and elimination along the gut–liver axis. Enrichment of microbial functions capable of modifying bile acid pools may therefore contribute to impaired bilirubin clearance and its accumulation in the circulation. In parallel, carbohydrate-active enzyme (CAZy) analysis demonstrated group-specific differences in glycoside hydrolases and glycosyltransferases, suggesting a remodeled capacity for carbohydrate utilization that could influence intestinal redox balance, mucosal integrity, and host–microbe signaling (Wardman et al., 2022). These functional alterations are broadly consistent with experimental and clinical evidence indicating that gut microbes modulate bile acid composition and enterohepatic circulation, underscoring the functional relevance of the microbiome in neonatal bilirubin homeostasis.

To move beyond observational associations, we employed bidirectional Mendelian randomization to explore potential causal relationships between gut microbiome features and neonatal jaundice. In the forward analyses, genetically predicted variation in several microbial metabolic pathways was associated with jaundice risk, whereas other pathways and selected Proteobacteria-related traits showed inverse associations. These findings suggest that inherited determinants influencing specific microbial functions may modulate susceptibility to neonatal jaundice, potentially through effects on bilirubin processing or gut–liver signaling. In contrast, the reverse Mendelian randomization analyses provided little evidence that genetic liability to neonatal jaundice exerts a substantial effect on gut microbiome composition or functional pathways. While Mendelian randomization strengthens causal inference by reducing confounding and reverse causation, these results should be interpreted cautiously in light of potential pleiotropy, measurement error, and differences between the GWAS source populations and our neonatal cohort (Richmond and Davey Smith, 2022). Accordingly, we interpret our Mendelian randomization findings as supportive—rather than definitive—evidence for a contributory role of the gut microbiome in neonatal jaundice.

Despite the strengths of integrating deep shotgun metagenomic profiling with genetic causal inference, several limitations warrant consideration. First, the clinical cohort was relatively small and recruited from a single center, which may limit the generalizability of the observed microbial patterns; larger multicenter studies encompassing diverse ethnic and geographic populations are therefore needed. Second, the cross-sectional study design precludes assessment of temporal dynamics in the neonatal microbiome before, during, and after jaundice, and limits our ability to disentangle the effects of clinical interventions such as phototherapy, feeding practices, and antibiotic exposure (Ding et al., 2021). Third, the Mendelian randomization analyses relied on publicly available GWAS summary statistics derived predominantly from non-neonatal populations, and the genetic instruments captured only a modest proportion of variance in microbiome traits. Future longitudinal birth cohorts and randomized controlled trials of microbiome-targeted interventions—such as probiotics, prebiotics, or maternal dietary modulation—will be essential to validate these findings and to determine whether modulation of the infant gut microbiota can meaningfully contribute to the prevention or management of neonatal jaundice.

## 5 Conclusion

This study integrates shotgun metagenomic profiling and bidirectional Mendelian randomization to investigate the relationship between neonatal jaundice (NJ) and the gut microbiome. We identified distinct differences in gut microbiota composition and functional potential between jaundiced and healthy neonates, particularly involving pathways related to bile acid metabolism, carbohydrate utilization, and energy metabolism. These findings suggest that alterations in the gut microbiome are closely associated with neonatal jaundice and may play a role in bilirubin metabolism.

Genetic evidence from Mendelian randomization analyses provides supportive—but not definitive—evidence that specific microbial functional pathways may contribute to susceptibility to neonatal jaundice, while offering limited support for reverse causality from jaundice to gut microbiome traits. Together, these results highlight a potential rationale for considering the gut microbiome as a modifiable factor in neonatal jaundice and underscore the need for larger, longitudinal, and interventional studies to clarify causal mechanisms and to evaluate the feasibility of microbiome-targeted strategies in neonatal care.

## Data Availability

The raw shotgun metagenomic sequencing data generated in this study have been deposited in the European Nucleotide Archive (ENA) under study accession number PRJEB108038 (secondary accession ERP188903).

## Supplementary material

Supplementary Figure S1 Overview of the study design and analytical workflow integrating shotgun metagenomic sequencing and bidirectional Mendelian randomization analysis.
