# Supplementary figures and images for: Neonatal jaundice and the infant gut microbiome: an integrated shotgun metagenomics and bidirectional Mendelian randomization study in Xinjiang
Source: Front Microbiol. 2026 Feb 26;17:1761712. doi: 10.3389/fmicb.2026.1761712 (PMC12980891; doi:10.3389/fmicb.2026.1761712)

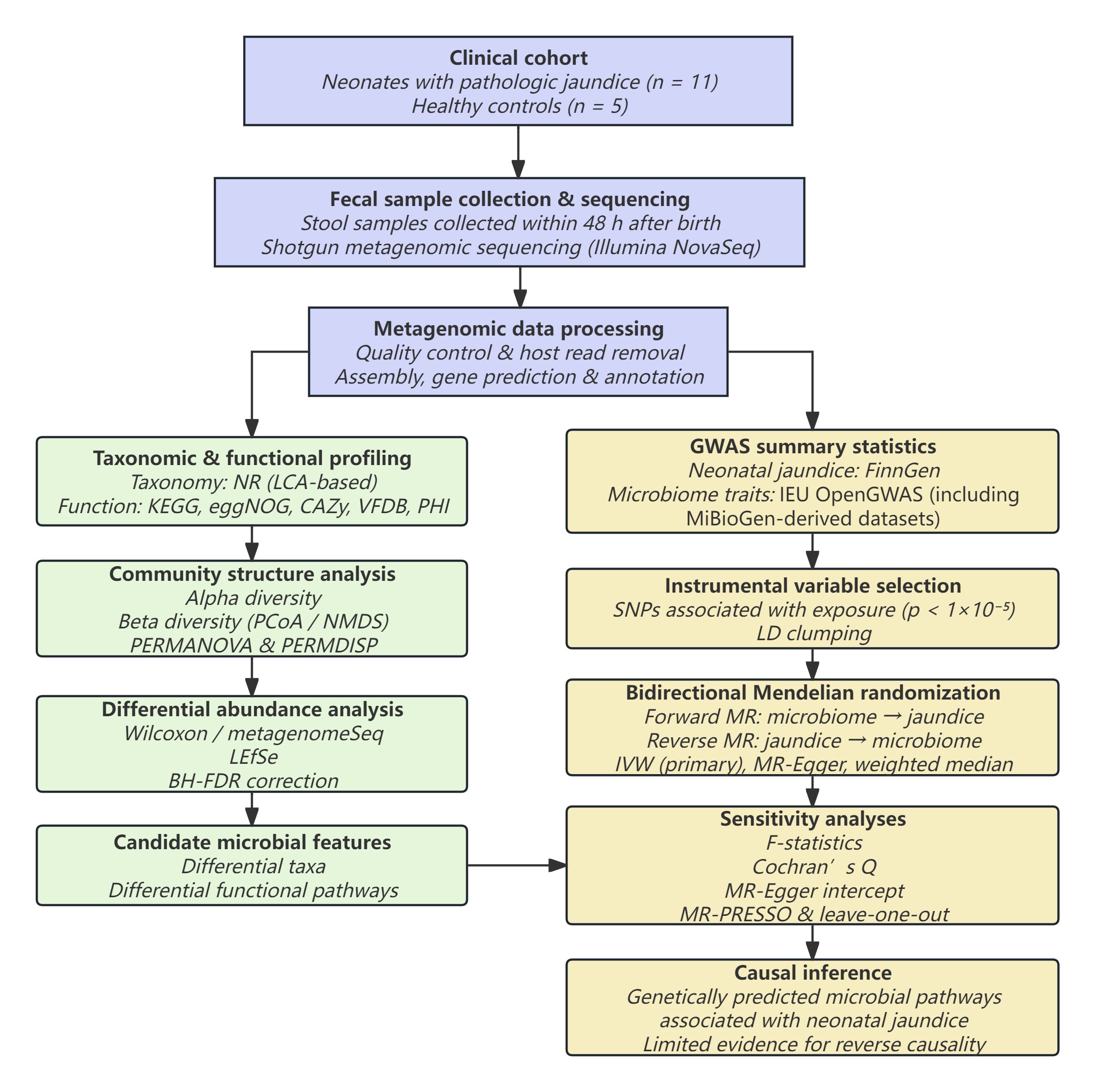

Supplement: Supplementary Figure S1 — Overview of the study design and analytical workflow integrating shotgun metagenomic sequencing and bidirectional Mendelian randomization analysis. [file Image_1.jpeg]
